# Building a cardiovascular disease observatory in primary care in Crete, Greece: design, implementation, and lessons learned for interdisciplinary digital surveillance

**DOI:** 10.3389/fcvm.2026.1906642

**Published:** 2026-10-30

**Authors:** Marilena Anastasaki, Foteini Anastasiou, Fragiskos Parthenakis, Christos Lionis

**Affiliations:** 1 Clinic of Social and Family Medicine, School of Medicine, University of Crete, Heraklion, Crete, Greece; 2 Cardiology Department, Heraklion University Hospital, Heraklion, Crete, Greece

**Keywords:** cardiovascular disease, digital health, observatory, primary care (MeSH), surveillance

## Abstract

**Background:**

Cardiovascular disease (CVD) remains the leading cause of mortality in Greece. Primary care is the appropriate setting for surveillance of risk factors and morbidity at the community level, with the nursing workforce being central to CVD care. However, CVD-specialist nurses are seldom embedded differentially in Greek primary care. In this context, the School of Medicine of the University of Crete and the Region of Crete launched a regional initiative to design and pilot a CVD observatory aimed at supporting digitally enabled, multidisciplinary primary care.

**Aim:**

The aim of this work is to describe the design and implementation of the Cardiovascular Disease Observatory in Primary Health Care (CVD-PHC Observatory) of Crete; to reflect on the barriers, facilitators, and practical lessons that emerged during its development; and analyse the design priorities that need to be met for future efforts to meaningfully involve the nursing workforce.

**Programmatic elements:**

The initiative comprised five sequential phases: (i) co-design of a structured CVD data collection instrument covering sociodemographic, behavioural, clinical, laboratory, biomarker, imaging, and treatment variables; (ii) development of a stand-alone digital recording platform with role-based access for data administrators and field recorders, supporting both real-time and asynchronous entry; (iii) recruitment and training of primary care practices across the four prefectures of Crete; (iv) 3-month pilot deployment, followed by structured feedback; and (v) subsequent 12-month deployment across the same sentinel network.

**Lessons learned:**

The platform was technically feasible and usable, with 600 patient records entered during the 12-month deployment. However, its long-term value was constrained by the lack of interoperability with national e-prescription and electronic health record systems, by the burden of duplicate data entry, and by the disruption of the COVID-19 pandemic. As no CVD nurses were embedded in the participating sites, deployment was delivered by general practitioners. Thus, the manuscript reports lessons learned for future interdisciplinary efforts rather than a nursing intervention. The initiative offers transferable assets for digital CVD surveillance, interoperability, and the meaningful inclusion on cardiovascular nursing in regional health observatories elsewhere in southern Europe.

## 1 Introduction

Cardiovascular disease (CVD) is the leading cause of death in Europe and a major contributor to disability and premature mortality in Greece. The traditionally low CVD risk profile of the Cretan population, documented in the Seven Countries Study and confirmed in later regional cohorts, has changed substantially over the past three decades, with rising prevalence of obesity, metabolic syndrome, and dyslipidaemia, particularly among rural and ageing populations (1–3). A cross-sectional study by our group in primary care patients aged 40 years and older identified a prevalence of metabolic syndrome of approximately 74% and a substantial proportion of individuals with a 10-year Systematic COronary Risk Evaluation (SCORE) risk above 10%, indicating an urgent need for systematic surveillance and targeted action (3).

Primary healthcare (PHC) is internationally recognised as the most appropriate setting for population-level CVD surveillance and risk management (4, 5). However, primary care surveillance in Greece has historically been fragmented across paper records, hospital discharge data, and isolated electronic prescribing systems, with no integrated regional infrastructure that could support longitudinal monitoring of CVD morbidity, risk factors, or care quality. The recent acceleration of digital transformation in European primary care, including the expansion of Greece's national e-prescription system, has created new opportunities to embed structured CVD surveillance within routine primary care workflows (6, 7).

Internationally, the nursing workforce is increasingly central to CVD care in primary and community settings, where nurses are often the principal point of contact for chronic disease monitoring, patient education, and follow-up coordination. They are, also, increasingly expected to engage with digital tools that support risk stratification, behavioural counselling, and care continuity (8, 9). Designing CVD surveillance infrastructure that can support the work of interdisciplinary primary care teams, including cardiovascular nurses, is therefore a priority for digitally enabled cardiovascular care.

In 2019, the School of Medicine at the University of Crete and the Region of Crete collaborated to develop the Cardiovascular Disease Observatory in Primary Health Care (CVD-PHC Observatory) of Crete, envisaged as a regional big-data infrastructure for the systematic recording of CVD morbidity, risk factors, treatments, and outcomes among primary care attendees. The present article describes the design, implementation, and pilot deployment of this initiative and reflects on the practical, organisational, and methodological lessons that emerged. In Greece, although CVD nursing specialisation is formally recognised, it is not differentiated in the deployment of the nursing workforce within primary care, and team-based care remains the exception despite the ongoing primary care reform. Thus, our initiative was delivered without formal involvement of cardiovascular nurses, as they were not embedded in our sites. We discuss this gap, use emerging lessons to identify design priorities that would be needed for future digital surveillance infrastructure to meaningfully involve and support the nursing workforce, and promote multidisciplinary CVD care within primary care.

## 2 Context

### 2.1 Setting

Crete is the largest island of Greece located in the southernmost region of the European Union, with approximately 624,000 permanent residents distributed across four prefectures (Heraklion, Chania, Rethymno, and Lasithi). The island combines a major urban centre (Heraklion) with extensive rural and mountainous areas that pose well-documented challenges for healthcare access, continuity, and surveillance (10). Primary care on the island is delivered through a network of public health centres, satellite rural practices, and private general practitioners (GPs), operating under the umbrella of the Regional Health Authority. Crete has 22 public health centres in rural and semi-urban areas, with the prefecture of Heraklion having the largest concentration (with seven health centres), followed by Chania (six), Rethymno (five), and Lasithi (four). There are also 129 satellite rural practices affiliated to these health centres (Heraklion 47, Chania 38, Rethymno 22, and Lasithi 22) and four urban health centres (one in each prefecture). The network is not proportional to population, as rural and remote areas have dedicated centres despite relatively small populations. The regional health authorities publish “population of responsibility” figures, which sum to approximately 594,600 people, amounting to approximately 33,000 people per centre on average. However, it should be clearly noted that this is a catchment population, not the number of people who actually visit the centre during a year. Consequently, those figures are useful for measuring territorial coverage, but not actual utilisation. Despite the ongoing primary care reform, team-based care is still lagging behind in Crete and Greece.

### 2.2 Population and rationale for site selection

The CVD-PHC Observatory was designed to enrol primary care attendees aged 18 years and older with at least one confirmed international classification of diseases (ICD)-10 diagnosis of cardiovascular disease (codes I10–I16 hypertensive diseases, I20–I25 ischaemic heart disease, I30–I52 other forms of heart disease, I60–I69 cerebrovascular disease, I70–I79 diseases of arteries, and I95–I99 other disorders of the circulatory system). Ten sentinel primary care sites were purposively selected from areas of elevated CVD burden, as identified by the health maps of the Regional Health Authority of Crete and by prior epidemiological work conducted by our group in Cretan PHC populations (1, 3, 11). Purposive selection was chosen rather than formal cut-off thresholds because the small number of sentinel sites (*n* = 10) and the exploratory character of the initiative did not warrant a threshold-based sampling frame or a population representative sample. The 10 selected sites comprised 7 health centres (32% of the island's 22 rural/semi-urban health centres), 2 satellite rural practices, and 1 private general practice, distributed across all 4 prefectures.

### 2.3 Team and governance

The initiative was a collaboration between the Clinic of Social and Family Medicine (School of Medicine, University of Crete), the Cardiology Department of the University Hospital of Heraklion, and the Region of Crete (Regional decision 37/07-12-2018). Ethical approval was granted by the Research Ethics Committee of the University of Crete (No. 41/21.02.2019 for phases 1–4 and No. 108/13.09.2021 for phase 5).

## 3 Key programmatic elements

The CVD-PHC Observatory was implemented in five sequential phases between early 2019 and late 2020. Figure 1 summarises the overall workflow.

**Figure 1:**
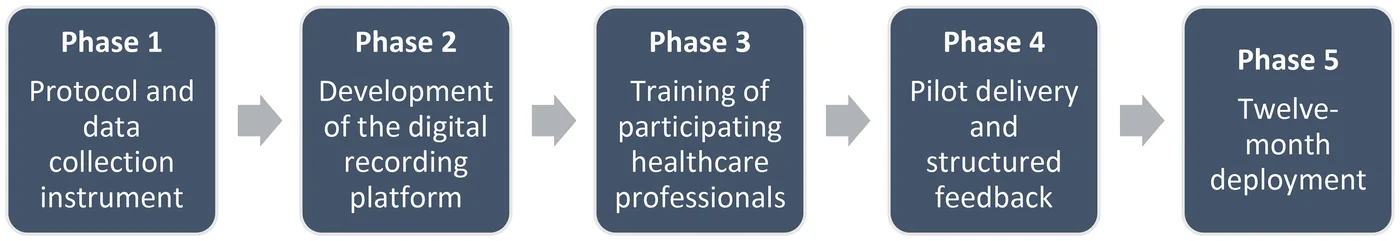
Programme flow diagram.

### 3.1 Phase 1: co-design of the research protocol and the structured data collection instrument

The first phase (early to mid-2019) produced a written research protocol and a structured data collection instrument (registration form). The structured instrument was developed by a multidisciplinary working group of general practitioners, epidemiologists, and public health researchers from the School of Medicine (University of Crete) and cardiologists from the Cardiology Department of the University Hospital of Heraklion. Domain selection was informed by European guidelines on cardiovascular risk assessment and management in primary care (SCORE-based risk stratification), literature, and our group's prior experience in Cretan primary care (3, 11). Individual variables within each domain were selected on the basis of their routine availability in Greek primary care records. Two rounds of working group review and feedback were performed, with the final version covering 10 domains: (1) cardiovascular diagnosis at entry (ICD-10 coded), hospitalisations and complications, discharge, and current treatment [anatomical therapeutic chemical (ATC)-coded]; (2) sociodemographic characteristics including insurance status, residence, and household income; (3) lifestyle behaviours including smoking, alcohol, physical activity, and Mediterranean-style dietary intake; (4) family history of CVD; (5) personal medical history including hypertension, diabetes, dyslipidaemia, thyroid disorders, chronic obstructive pulmonary disease (COPD), renal insufficiency, and malignancy; (6) anthropometric and vital signs; (7) laboratory results (lipid profile, fasting glucose, HbA1c, liver and kidney function, and full blood count); (8) cardiac biomarkers [B-type natriuretic peptide (BNP)/pro–B-type natriuretic peptide (proBNP), troponins, creatine kinase-MB (CK-MB), soluble urokinase plasminogen activator receptor (SuPAR), C-reactive protein (CRP), and lipoprotein-associated phospholipase A2 assay (Plac test)]; (9) electrocardiography and imaging findings (left ventricular hypertrophy, ejection fraction, CT findings); (10) other current pharmacotherapy (ATC-coded); and (11) new incident cardiovascular events recorded longitudinally.

A patient information and consent form was developed in plain Greek, explaining purposes, data handling, and the right to withdraw, in compliance with the Greek implementation of general data protection regulation (GDPR).

### 3.2 Phase 2: development of the digital recording platform

The platform was developed as a Windows desktop application using C++ with Windows Forms, and a Microsoft Access back end was embedded in the application (mid 2019 to early 2020). Each installed instance held a local database and could synchronise upward to a central project database. Two user roles were defined:

□ A recorder role with rights restricted to entering new patient records
□ An administrator role with rights to retrieve and edit existing records, upload them to the central database, create new users, and curate controlled vocabularies (notably the ICD-10 picklist)

Records could be saved in draft and resumed later. To reduce data entry errors and burden, the form was built around constrained picklists and conditional fields wherever possible, with free text reserved for clearly unstructured items. Identifying data (name, social security number) were intended to be encrypted at the application level and segregated from the analytic dataset; differential access rights were enforced through the role model.

Although our original idea envisaged a relational big-data architecture using SQL-based programming, the aforementioned technology stack was selected due to pragmatic constraints. These included offline operability in rural primary care sites with unreliable connectivity, the absence of infrastructure requirements on the practice side beyond a Windows workstation, the limited development and licensing cost within the study budget, and the absence at the time of a national interoperability framework that would have made a more advanced architecture feasible and usable in Greek primary care, a large part of which is offered in rural and remote areas. Screenshots of the digital recording platform (available in Greek only) are provided in the Supplementary Material.

### 3.3 Phase 3: training of participating healthcare professionals

Ten GPs and their primary care teams of nurses and other professionals were invited to act as sentinel sites; they were selected to cover the four prefectures of Crete and to include practices in areas of high CVD burden.

A 2-hour structured training session was delivered at the Clinic of Social and Family Medicine in Heraklion, with the option of remote attendance (July 2020). Six of the 10 participating sites attended (5 in person, 1 remotely). The session combined a live demonstration of the platform in a real-world workflow with a moderated focus group discussion structured around three themes: (a) adequacy of the dataset; (b) functionality of the system; and (c) practicality for everyday clinical use. Participants were provided with written guidance on patient selection, motivational interviewing techniques for invitation, and the informed consent procedure. Following the session, GPs were asked to use the platform in pilot mode and to report any difficulties. The sites that did not attend the session were trained individually, through one-to-one videoconference sessions. These individual sessions used the same two-hour training structure as the central session (live demonstration of the platform followed by a moderated discussion around dataset adequacy, system functionality, and clinical practicality), adapted only in that the focus group element of the central session was replaced by a one-to-one structured discussion.

### 3.4 Phase 4: pilot deployment and structured feedback

Following training, GPs used the platform for approximately 3 months (August–October 2020). The pilot was specifically designed to elicit usability and integration feedback rather than to generate a representative epidemiological dataset. At the end of the pilot, GPs were invited to submit structured comments on platform improvements, which were then synthesised into a set of recommendations intended to inform a subsequent generation of the platform (Box 1).

BOX 1 **Pilot feedback from sentinel general practitioners.**

**Table:** 

1. Interoperability with existing systems, in particular national e-prescription and any electronic health record systems used in the practice. 2. Reduction in the number of fields, to shorten recording time and reduce duplication with routine care. 3. ICD-10 coding embedded throughout the form (not only at the entry diagnosis), with an open-text fallback when the code cannot be located. 4. Ability to update the same record across multiple encounters (this had been disabled in the pilot for data protection reasons, since identifying data were not retained). 5. Multiple concurrent ICD-10 cardiovascular diagnoses per patient, captured across different encounters. 6. Upgrade to a “smart” version of the platform supporting attachments (.pdf, .jpg, .mp4) such as laboratory results and specialist letters. 7. Export of de-identified results not only in coded Excel format, but also in human-readable Excel and PDF. 8. Restructuring of the “entry diagnosis” variable so that it captures the cardiovascular ICD-10 code rather than the source of the diagnosis (hospital, PHC, other), which should be a separate variable. 9. Conversion of yes/no fields with downstream items into a conditional drop-down menu pattern.

GPs reported no concerns regarding the speed or reliability of the platform itself and assessed the recording workflow as comprehensible. The main collective recommendation was that any future iteration must be integrated with—rather than parallel to—the systems clinicians already use in routine practice.

### 3.5 Phase 5: 12-month deployment

Following the 2020 pilot, an upgraded version of the platform was deployed across the same sentinel network. The upgrade addressed items 3, 4, 5, 7, 8, and 9 of the pilot feedback (Box 1), namely, recommendations on ICD-10 coverage, multi-encounter record updates, multiple concurrent diagnoses per patient, extended export formats, restructuring of the entry diagnosis variable, and conditional drop-down fields, retaining the 10-domain structure and underlying architecture. More substantive changes requiring architectural shift, such as interoperability with the national e-prescription and electronic health record systems, remained beyond project budget.

Participating sites entered patient records over the subsequent 12 months (December 2021–December 2022), with interim progress reports collected to resolve difficulties and monitor recording. Eligible patients were identified by participating GP during routine consultations. For those who consented, recording was completed either within the same consultation or, where time was insufficient, at a dedicated appointment. Mean time to complete the full record was approximately 15–20 min. During this deployment, the ten sites entered a total of 600 patient records. A detailed epidemiological analysis of this dataset is beyond the scope of the present report and will be reported in separate papers. However, a summary of the sociodemographic, clinical, and behavioural profile of the recorded population is provided in Table 1.

**Table 1:** Sociodemographic, clinical, and behavioural profile of the recorded population during the 2021–2022 12-month deployment phase of the CVD-PHC observatory (*N* = 600).

Characteristic	n/mean	%/SD
Age (years, mean and SD)	65.1	9.7
Sex (women)	325	54.2
Body mass index (kg/m^2^, mean and SD)	28.9	4.3
Waist circumference (cm, mean and SD)	98.7	13.2
Systolic blood pressure (mmHg, mean and SD)	129.5	14.2
Diastolic blood pressure (mmHg, mean and SD)	79.8	9.9
Smoking status (current/ex-smoker)	300	49.9
Entry diagnosis (hypertensive diseases I10–I16)	250	41.7
Cardiovascular comorbidities (dyslipidaemia)	455	75.8
Family history of cardiovascular disease (yes)	421	70.2

For multi-category variables, only the most frequent categories are shown.

The deployment confirmed the technical feasibility and usability of the platform under real-world primary care conditions and generated a first snapshot of the cardiovascular risk profile of the recorded population across urban and rural Crete. It did not, however, resolve the underlying interoperability limitations identified in the pilot phase, which are discussed further in the following sections.

## 4 Discussion

### 4.1 What worked

The CVD-PHC Observatory established several elements that we consider transferable. First, a structured, ICD-10- and ATC-coded data dictionary tailored to CVD primary care in a Mediterranean setting was successfully co-developed and approved by an ethics committee and the regional data protection process; this instrument remains available and could be repurposed for future surveillance work. Second, a role-based digital recording tool was developed, deployed, and used in a real-world primary care environment without significant technical failure. Third, a multi-prefecture network of sentinel GPs spanning urban and rural Crete was successfully constituted, demonstrating the feasibility of region-wide engagement under modest funding. Fourth, the project generated implementation knowledge that constitutes a usable asset for a next-generation tool.

### 4.2 What did not work, and why

The initial framing of the platform as a big-data infrastructure was not fully realised. In practice, the delivered system was a structured registry tool, rather than a big-data platform in the technical sense (no linkage to administrative or hospital datasets, no real-time analytics, no automated data quality monitoring, and no large-volume data ingestion). Failures of large-scale primary care digital health implementation attributable to inadequate infrastructure, connectivity, and interoperability have been documented in national electronic health record system (EHRS) deployment attempts in other countries (12), and interoperability, legal fragmentation, and security remain persistent challenges even at the EU level, as recently documented by the HealthData@EU Pilot experience (13). Similarly, in our case, several factors contributed to the gap between ambition and outcome, including technical, legislative and funding issues.

#### 4.2.1 Scope and budget asymmetry

The budget was sufficient to design a protocol, build a single-tier desktop application, and run a small pilot, but not to deliver a federated, interoperable, multi-source big-data infrastructure of the kind the original protocol envisaged. Acknowledgement of this asymmetry is, in our view, one of the most important lessons of the project for future regional health observatories. Surveillance infrastructure that is technically big data requires investment in interoperability layers, data engineering capacity, and ongoing data stewardship that go well beyond the resources typically available for a single deliverable-based regional contract (14).

#### 4.2.2 Lack of interoperability with national systems

The pilot GPs were unanimous that any system requiring duplicate data entry in parallel to their existing electronic prescribing and medical record systems would not be sustainable in routine care. This has been also reported in other similar published initiatives (6), while European work on cross-border patient summary exchange has also demonstrated that meaningful interoperability requires deep alignment on shared clinical document standards that no single regional deployment can produce alone (15). Our CVD-PHC Observatory platform was designed as a stand-alone application precisely because the national landscape at the time did not support interoperable integration. As such, this design choice ensured deliverability of the pilot but limited its scalability. Any next-generation iteration of the CVD-PHC Observatory should migrate to a modern web-based architecture to enable, among others, bidirectional exchange with the national e-prescription system and future electronic health record platforms, under appropriate data protection regulations.

#### 4.2.3 GDPR-compliant identifier management for longitudinal linkage

A specific challenge for any future effort is the reconciliation of GDPR-compliant identifier management with longitudinal patient linkage that meaningful CVD surveillance requires (16). Our deployment avoided this by disabling multi-encounter updates of the same record. A federated next-generation Observatory would need to adopt a pseudonymisation architecture in which a stable, one-way-hashed patient identifier permits within-patient longitudinal linkage across encounters and sites without exposing directly identifying data to analysts. The identifier-generation function should reside with an independent institutional trusted third party, with strict role-based access separating the identifier layer from the analytic layer, aligned with GDPR regulations (17).

#### 4.2.4 External discontinuity (COVID-19 pandemic)

Training was delivered in July 2020, in the middle of the first year of the COVID-19 pandemic, which absorbed primary care capacity in Greece and globally and disrupted routine chronic disease surveillance internationally (18). The data collected during the pilot, intended to inform refinement rather than to generate population estimates, remained accessible only for a short period of time as per protocol and contract requirements. It is a real-world feature of small-scale, time-limited regional initiatives that data stewardship arrangements rarely persist beyond the contract itself, and should be explicitly addressed by future observatories through institutional data hosting and long-term stewardship agreements.

#### 4.2.5 Workforce

Due to lack of an established team-based culture within local primary care units, the pilot was delivered primarily by GPs as data recordings. Although they were explicitly instructed and urged to engage their co-working nurses and other professionals, this was not always possible. As such, the role of nurses and other primary care staff in CVD surveillance and management was under-represented. This is particularly important considering that, although acknowledged as a nursing specialisation, CVD nurses are seldom embedded in PHC in Greece. Across Europe, cardiovascular nursing is increasingly central to risk factor assessment, lifestyle counselling, medication monitoring, and patient education in primary and community care (8, 19). Future iterations of the Observatory should be co-designed with the nursing workforce, with clear delineation of which fields are routinely captured by nurses, which by physicians, and how the system supports, rather than duplicates, interdisciplinary workflows.

### 4.3 Implications for future nursing-inclusive digital CVD surveillance

Two forward-looking messages emerge for digital CVD nursing. Because cardiovascular nurses were not involved in the present initiative, the observations that follow represent priorities for future efforts rather than findings of a nursing intervention. First, digital CVD surveillance in primary care must be built around routine clinical workflows, not adjacent to them (20). In the Greek primary care context, this message could take the form of a formally defined split between fields routinely captured by nurses (anthropometry, vital signs, lifestyle behaviours, and self-reported adherence) and by physicians (diagnosis coding, pharmacotherapy, and laboratory and imaging interpretation), along with protected time in the nurses’ schedule for structured CVD recording and a role-specific view of the digital platform. Co-design with the nursing workforce, who often hold the operational knowledge of how risk factor data are gathered and acted upon in practice, is a precondition for sustainability (19, 21). The feasibility of such adjustments is, however, constrained by the fact that the cardiovascular nursing specialisation, though formally recognised in Greece, is not currently differentiated in the primary care staffing model. Second, small regional initiatives can play a useful role in generating implementation knowledge. The structured feedback we obtained from sentinel GPs maps closely onto well-established usability and interoperability frameworks for digital health interventions (22) and can inform larger national or regional initiatives.

### 4.4 Limitations

This is a reflective community case study of a single regional initiative, with the limitations inherent to this design. We report only aggregate patient-level information from the 2021–2022 deployment (Table 1) as this report was intended for feasibility assessment rather than an epidemiological analysis which will be reported separately. Patient invitation, consent, and refusal rates were not systematically recorded, so denominator-based analysis and participation assessment cannot be performed. In addition, the qualitative feedback from participating GPs was synthesised rather than analysed through formal qualitative methods. Furthermore, we were unable to reconstruct the exact distribution of records as per site or field-level completeness metrics, which represents a data-stewardship limitation. In spite of the wave of nursing-led digital CVD interventions emerging in Europe (9, 15), CVD nurses are not currently deployed in a differentiated way within Greek primary care, due to which we could not explicitly involve them in our initiative. Generalisability to other settings should, finally, consider the specific organisational and regulatory features of Greek primary care.

## 5 Conclusions and next steps

The CVD-PHC Observatory of Crete demonstrates that a region-wide CVD surveillance instrument and digital recording platform can be co-designed, ethically approved, and deployed in primary care under modest regional funding. It also demonstrates the constraints that any sustainable next iteration should address, including the gap between big data ambition and funding, the centrality of interoperability, and the workforce integration barriers that no amount of platform refinement can resolve internally. Future regional CVD observatories should be explicitly built on modern web-based interoperable architecture, with institutional long-term data stewardship embedded from the outset, co-designed with the full primary care team, including CVD nursing, starting from the first phase rather than retrospectively.

## Data Availability

The raw data supporting the conclusions of this article will be made available by the authors without undue reservation.

## References

[1] Lindholm LH Koutis AD Lionis CD Vlachonikolis IG Isacsson A Fioretos M. Risk factors for ischaemic heart disease in a Greek population. A cross-sectional study of men and women living in the village of Spili in Crete. Eur Heart J. (1992) 13(3):291–8. 10.1093/oxfordjournals.eurheartj.a060165 1597213

[2] Filippidis FT Schoretsaniti S Dimitrakaki C Vardavas CI Behrakis P Connolly GN. Trends in cardiovascular risk factors in Greece before and during the financial crisis: the impact of social disparities. Eur J Public Health. (2014) 24(6):974–9. 10.1093/eurpub/cku028 24614651

[3] Anastasaki M Papadakis S Linardakis M Anyfantakis D Symvoulakis EK Lionis C. Cretan primary care research group. Burden of metabolic syndrome among primary care patients in Crete, Greece: a descriptive study. Eur J Gen Pract. (2020) 26(1):166–74. 10.1080/13814788.2020.1851676 33320035 PMC7744156

[4] Wändell PE De Waard A-KM Holzmann MJ Gornitzki C Lionis C De Wit N. Barriers and facilitators among health professionals in primary care to prevention of cardiometabolic diseases: a systematic review. Fam Pract. (2018) 35(4):383–98. 10.1093/fampra/cmx137 29385438

[5] Ludt S Van Lieshout J Campbell SM Rochon J Ose D Freund T. Development and pilot of an internationally standardized measure of cardiovascular risk management in European primary care. BMC Health Serv Res. (2011) 11:70. 10.1186/1472-6963-11-70 21473758 PMC3080793

[6] Piera-Jiménez J Dedeu T Pagliari C Trupec T Stoffers J Bavafa H. Strengthening primary health care in Europe with digital solutions. Aten Primaria. (2024) 56(10):102904. 10.1016/j.aprim.2024.102904 PMC11070233 38692228

[7] Kharlamov AN Lamberts M. Digital medicine: the next big leap advancing cardiovascular science. BMC Cardiovasc Disord. (2023) 23:25. 10.1186/s12872-022-02971-5 36650433 PMC9847174

[8] Bulto LN Roseleur J Noonan S Pinero de Plaza MA Champion S Dafny HA. Effectiveness of nurse-led interventions versus usual care to manage hypertension and lifestyle behaviour: a systematic review and meta-analysis. Eur J Cardiovasc Nurs. (2024) 23(1):21–32. 10.1093/eurjcn/zvad040 37130339

[9] Redfern J Coorey G Mulley J Scaria A Neubeck L Hafiz N. A digital health intervention for cardiovascular disease management in primary care (CONNECT): a randomised controlled trial. NPJ Digit Med. (2020) 3:117. 10.1038/s41746-020-00325-z 32964140 PMC7484809

[10] Lionis C Petelos E. The impact of the financial crisis on the quality of care in primary care: an issue that requires prompt attention. Qual Prim Care. (2013) 21(5):269–73. 24119512

[11] Anyfantakis D Symvoulakis EK Panagiotakos DB Tsetis D Castanas E Shea S. Impact of religiosity/spirituality on biological and preclinical markers related to cardiovascular disease. Results from the SPILI III Study. Hormones (Athens). (2013) 12(3):386–96. 10.1007/BF03401304 24121380

[12] Alzghaibi H Hutchings HA. Barriers to the implementation of large-scale electronic health record systems in primary healthcare centers: a mixed-methods study in Saudi Arabia. Front Med (Lausanne). (2025) 12:1516714. 10.3389/fmed.2025.1516714 40303363 PMC12037483

[13] Christiansen CF Doupi P Schutte N Ivanković D. Piloting an infrastructure for the secondary use of health data: learnings from the HealthData@EU pilot. Eur J Public Health. (2025) 35(Supplement_3):iii3–4. 10.1093/eurpub/ckaf073 40926476 PMC12420900

[14] González-García J Estupiñán-Romero F Tellería-Orriols C González-Galindo J Palmieri L Faragalli A. Coping with interoperability in the development of a federated research infrastructure: achievements, challenges and recommendations from the JA-InfAct. Arch Public Health. (2021) 79(1):221. 10.1186/s13690-021-00731-z PMC8656001 34879872

[15] Chronaki Catherine J Estelrich Ana F Cangioli Giorgio C Melgara Marcello J Kalra Dipak L Gonzaga Zabrina A. Interoperability standards enabling cross-border patient summary exchange. Stud Health Technol Inform. (2014) 205:256–60. 10.3233/978-1-61499-432-9-256 25160185

[16] European Data Protection Board. Guidelines 01/2025 on Pseudonymisation. Brussels: EDPB (2025). Available online at: https://www.edpb.europa.eu/ (Accessed May 11, 2026).

[17] Wündisch E Hufnagl P Brunecker P Meier zu Ummeln S Träger S Kopp M. Development of a trusted third party at a large university hospital: design and implementation study. JMIR Med Inform. (2024) 12:e53075. 10.2196/53075 PMC11040164 38632712

[18] Helal A Shahin L Abdelsalam M Ibrahim M. Global effect of COVID-19 pandemic on the rate of acute coronary syndrome admissions: a comprehensive review of published literature. Open Heart. (2021) 8(1):e001645. 10.1136/openhrt-2021-001645 34083389 PMC8182753

[19] Jin K Khonsari S Gallagher R Gallagher P Clark AM Freedman B. Telehealth interventions for the secondary prevention of coronary heart disease: a systematic review and meta-analysis. Eur J Cardiovasc Nurs. (2019) 18(4):260–71. 10.1177/1474515119826510 30667278

[20] Robles-Aguilar P Ruiz-Fernández MD Bermudo-Fuenmayor S. Digital health experiences of primary care nurses: a qualitative meta-synthesis. Int Nurs Rev. (2025) 72(3):e70069. 10.1111/inr.70069 40709747 PMC12291427

[21] Su JJ Yu DSF Paguio JT. Effect of eHealth cardiac rehabilitation on health outcomes of coronary heart disease patients: a systematic review and meta-analysis. J Adv Nurs. (2020) 76(3):754–72. 10.1111/jan.14272 31769527

[22] Joseph-Williams N Abhyankar P Boland L Bravo P Brenner AT Brodney S. What works in implementing patient decision aids in routine clinical settings? A rapid realist review and update from the international patient decision aid standards collaboration. Med Decis Making. (2021) 41(7):907–37. 10.1177/0272989X20978208 33319621 PMC8474331

