# Supplementary material for: Building a cardiovascular disease observatory in primary care in Crete, Greece: design, implementation, and lessons learned for interdisciplinary digital surveillance
Source: Front Cardiovasc Med. 2026 Oct 30;13:1906642. doi: 10.3389/fcvm.2026.1906642 (PMC13759269; doi:10.3389/fcvm.2026.1906642)
Supplement: Supplementary file 1 [file Datasheet1.pdf]

Supplementary File

Screenshots of the digital CVD recording platform developed in Crete, Greece

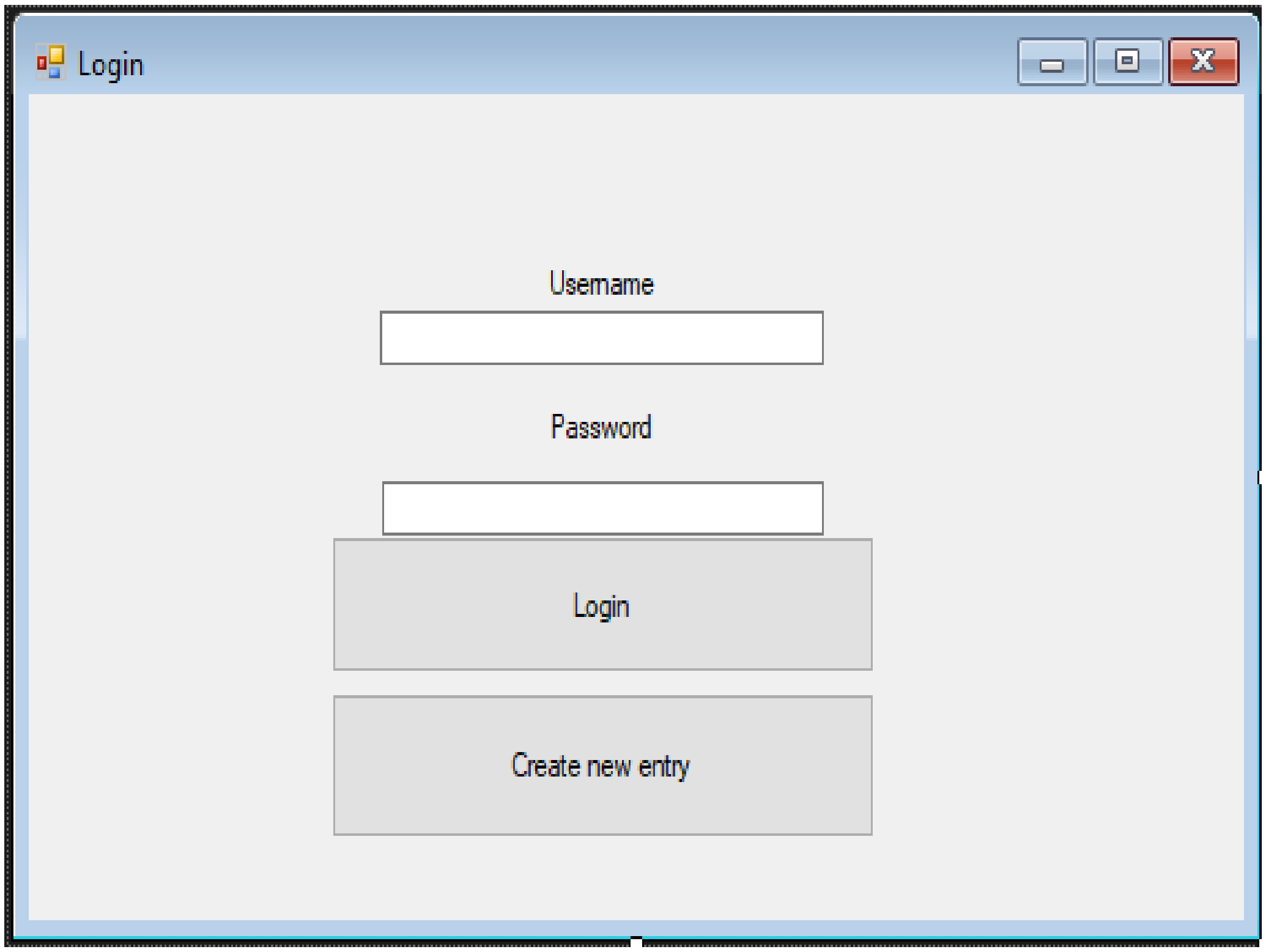

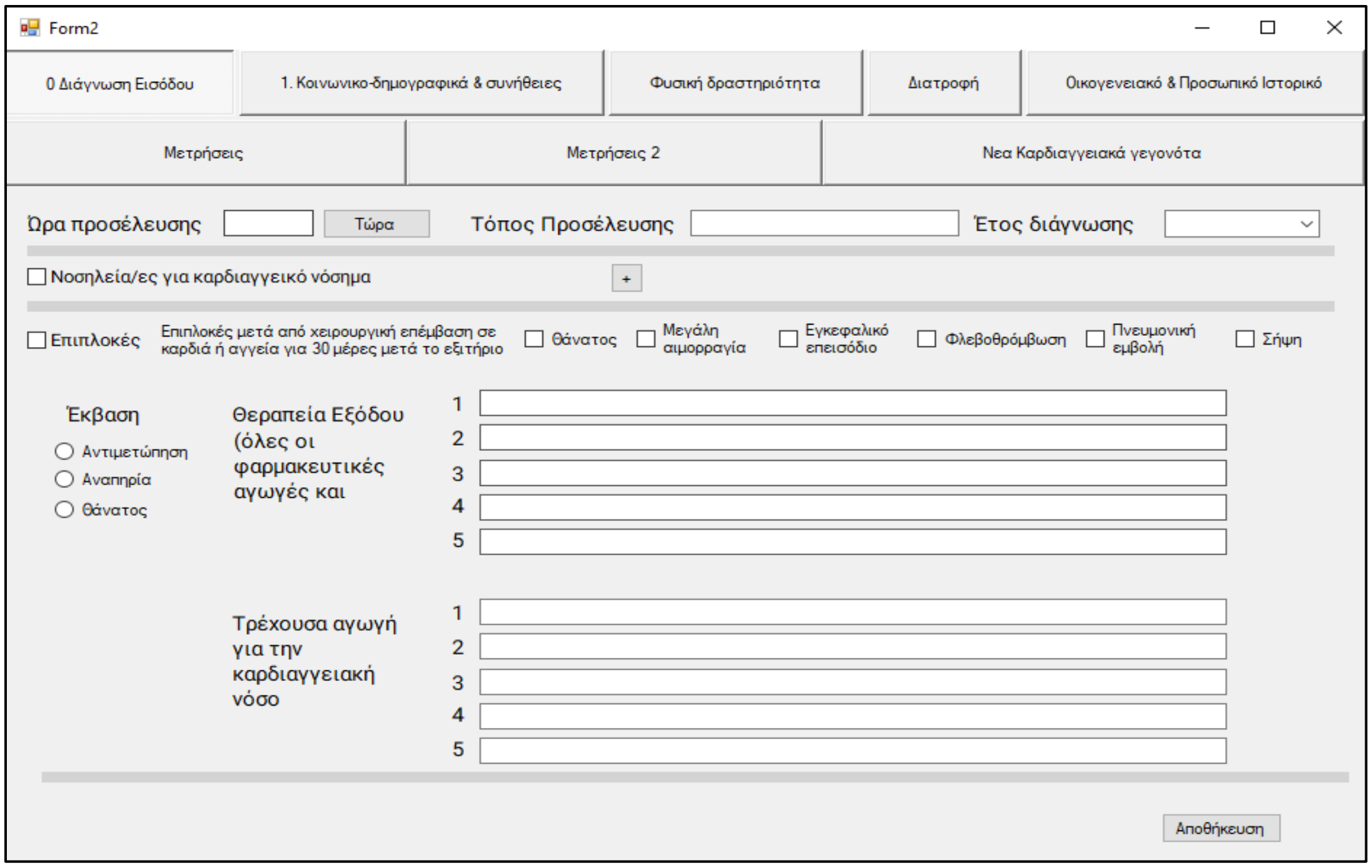

Form2
0 Διάγνωση Εισόδου
1. Κοινωνικο-δημογραφικά & συνήθειες
Φυσική δραστηριότητα
Διατροφή
Οικογενειακό & Προσωπικό Ιστορικό
Μετρήσεις
Μετρήσεις 2
Νεα Καρδιαγγειακά γεγονότα
Ώρα προσέλευσης
Τώρα
Τόπος Προσέλευσης
Έτος διάγνωσης
Νοσηλεία/ες για καρδιαγγειακό νόσημα
+
Επιπλοκές
Επιπλοκές μετά από χειρουργική επέμβαση σε καρδιά ή αγγεία για 30 μέρες μετά το εξιτήριο
Θάνατος
Μεγάλη αιμορραγία
Εγκεφαλικό επεισόδιο
Φλεβοθρόμβωση
Πνευμονική εμβολή
Σήψη
Έκβαση
Αντιμετώπηση
Αναπηρία
Θάνατος
Θεραπεία Εξόδου (όλες οι φαρμακευτικές αγωγές και
1
2
3
4
5
Τρέχουσα αγωγή για την καρδιαγγειακή νόσο
1
2
3
4
5
Αποθήκευση

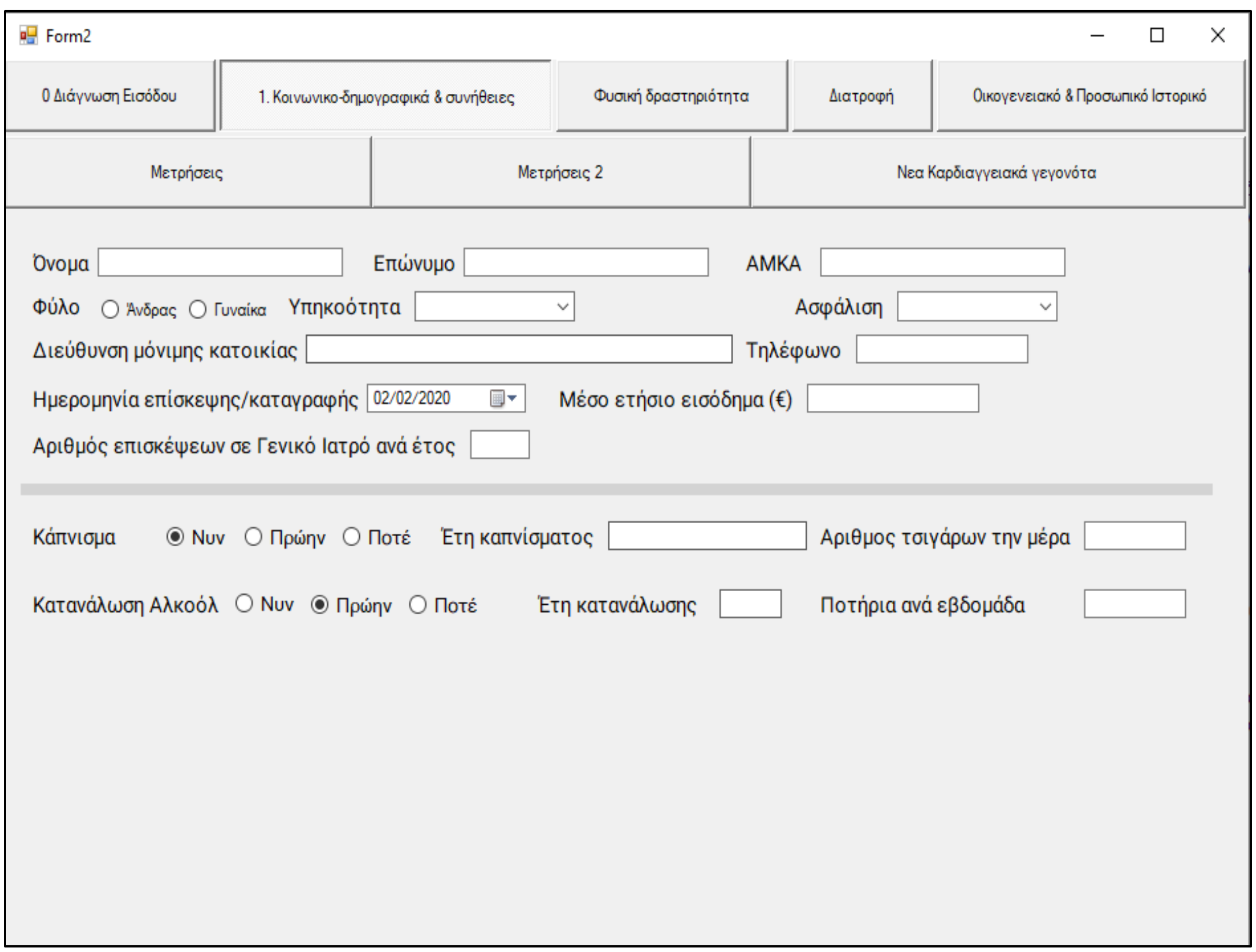

Form2
0 Διάγνωση Εισόδου
1. Κοινωνικο-δημογραφικά & συνήθειες
Φυσική δραστηριότητα
Διατροφή
Οικογενειακό & Προσωπικό Ιστορικό
Μετρήσεις
Μετρήσεις 2
Νεα Καρδιαγγειακά γεγονότα
Όνομα
Επώνυμο
ΑΜΚΑ
Φύλο
Άνδρας
Γυναίκα
Υπηκοότητα
Ασφάλιση
Διεύθυνση μόνιμης κατοικίας
Τηλέφωνο
Ημερομηνία επίσκεψης/καταγραφής
02/02/2020
Μέσο ετήσιο εισόδημα (€)
Αριθμός επισκέψεων σε Γενικό Ιατρό ανά έτος
Κάπνισμα
Νυν
Πρώην
Ποτέ
Έτη καπνίσματος
Αριθμος τσιγάρων την μέρα
Κατανάλωση Αλκοόλ
Νυν
Πρώην
Ποτέ
Έτη κατανάλωσης
Ποτήρια ανά εβδομάδα

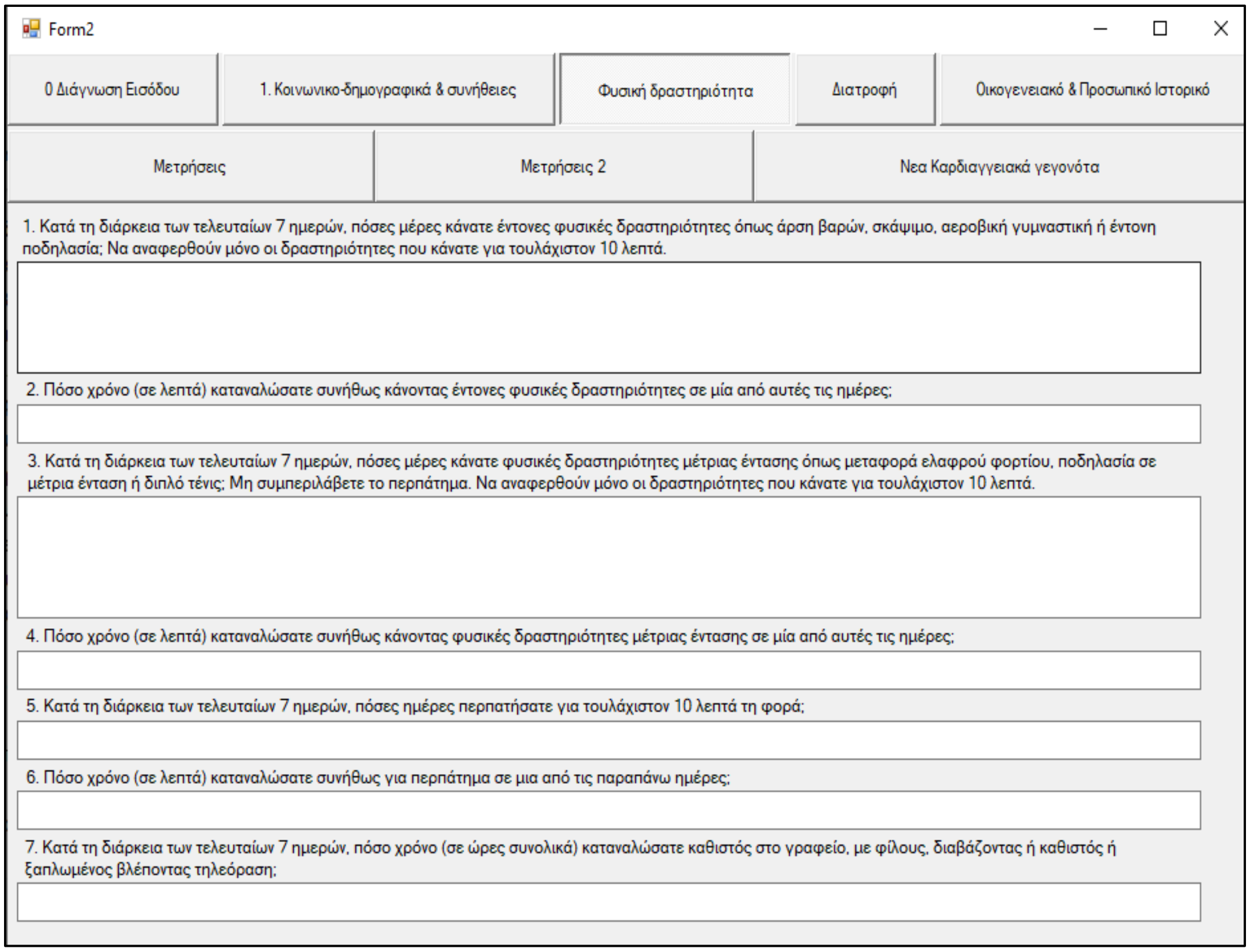

Form2

0 Διάγνωση Εισόδου | 1. Κοινωνικο-δημογραφικά & συνήθειες | Φυσική δραστηριότητα | Διατροφή | Οικογενειακό & Προσωπικό Ιστορικό

Μετρήσεις | Μετρήσεις 2 | Νεα Καρδιαγγειακά γεγονότα

1. Κατά τη διάρκεια των τελευταίων 7 ημερών, πόσες μέρες κάνατε έντονες φυσικές δραστηριότητες όπως άρση βαρών, σκάψιμο, αεροβική γυμναστική ή έντονη ποδηλασία; Να αναφερθούν μόνο οι δραστηριότητες που κάνατε για τουλάχιστον 10 λεπτά.

2. Πόσο χρόνο (σε λεπτά) καταναλώσατε συνήθως κάνοντας έντονες φυσικές δραστηριότητες σε μία από αυτές τις ημέρες;

3. Κατά τη διάρκεια των τελευταίων 7 ημερών, πόσες μέρες κάνατε φυσικές δραστηριότητες μέτριας έντασης όπως μεταφορά ελαφρού φορτίου, ποδηλασία σε μέτρια ένταση ή διπλό τένις; Μη συμπεριλάβετε το περπάτημα. Να αναφερθούν μόνο οι δραστηριότητες που κάνατε για τουλάχιστον 10 λεπτά.

4. Πόσο χρόνο (σε λεπτά) καταναλώσατε συνήθως κάνοντας φυσικές δραστηριότητες μέτριας έντασης σε μία από αυτές τις ημέρες;

5. Κατά τη διάρκεια των τελευταίων 7 ημερών, πόσες ημέρες περπατήσατε για τουλάχιστον 10 λεπτά τη φορά;

6. Πόσο χρόνο (σε λεπτά) καταναλώσατε συνήθως για περπάτημα σε μια από τις παραπάνω ημέρες;

7. Κατά τη διάρκεια των τελευταίων 7 ημερών, πόσο χρόνο (σε ώρες συνολικά) καταναλώσατε καθιστός στο γραφείο, με φίλους, διαβάζοντας ή καθιστός ή ξαπλωμένος βλέποντας τηλεόραση;

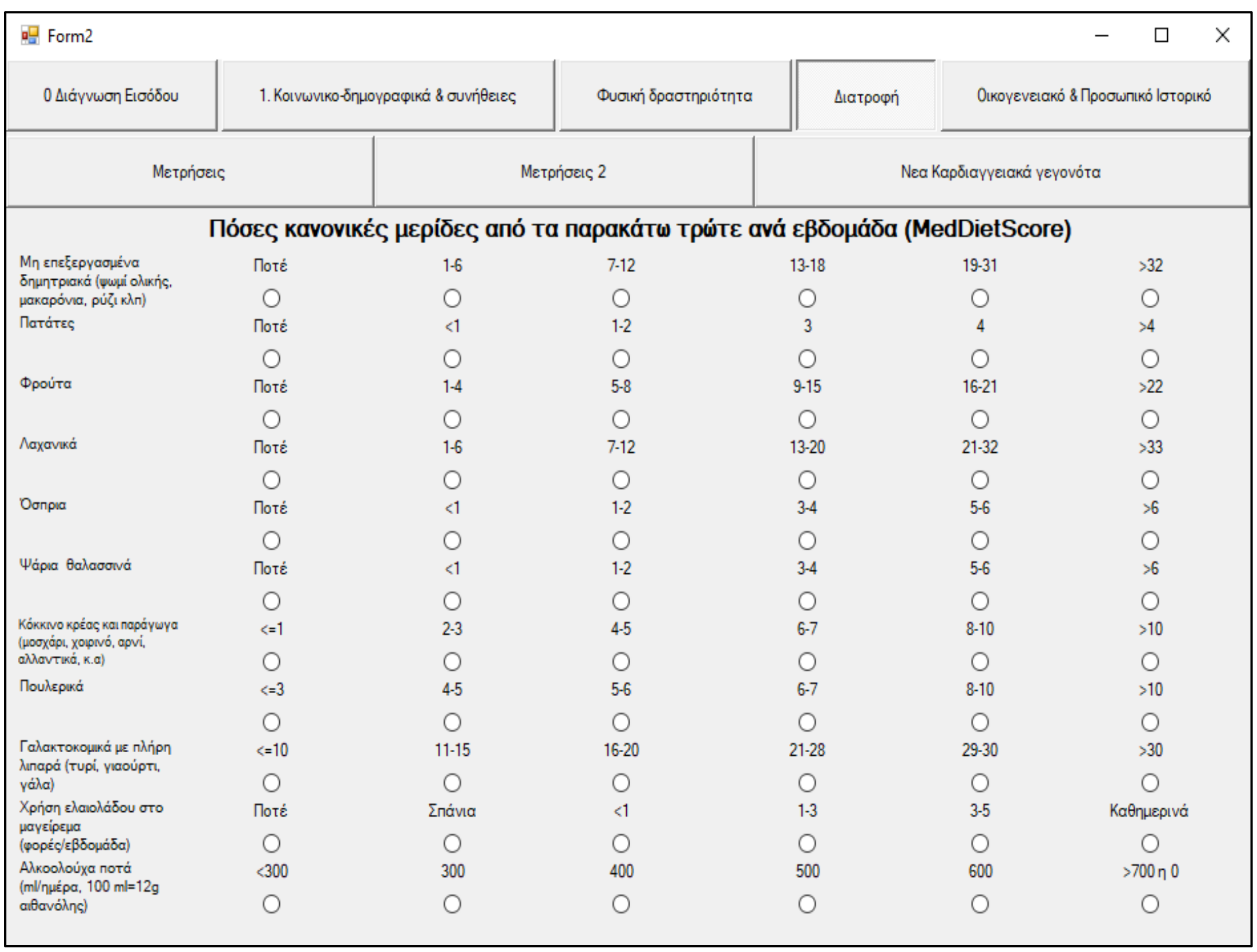

Form2

0 Διάγνωση Εισόδου | 1. Κοινωνικο-δημογραφικά & συνήθειες | Φυσική δραστηριότητα | Διατροφή | Οικογενειακό & Προσωπικό Ιστορικό

Μετρήσεις | Μετρήσεις 2 | Νεα Καρδιαγγειακά γεγονότα

Πόσες κανονικές μερίδες από τα παρακάτω τρώτε ανά εβδομάδα (MedDietScore)

Μη επεξεργασμένα δημητριακά (ψωμί ολικής, μακαρόνια, ρύζι κλπ)	Ποτέ	1-6	7-12	13-18	19-31	>32
Πατάτες	Ποτέ	<1	1-2	3	4	>4
Φρούτα	Ποτέ	1-4	5-8	9-15	16-21	>22
Λαχανικά	Ποτέ	1-6	7-12	13-20	21-32	>33
Όσπρια	Ποτέ	<1	1-2	3-4	5-6	>6
Ψάρια θαλασσινά	Ποτέ	<1	1-2	3-4	5-6	>6
Κόκκινο κρέας και παράγωγα (μοσχάρι, χοιρινό, αρνί, αλλαντικά, κ.α)	<=1	2-3	4-5	6-7	8-10	>10
Πουλερικά	<=3	4-5	5-6	6-7	8-10	>10
Γαλακτοκομικά με πλήρη λιπαρά (τυρί, γιαούρτι, γάλα)	<=10	11-15	16-20	21-28	29-30	>30
Χρήση ελαιολάδου στο μαγείρεμα (φορές/εβδομάδα)	Ποτέ	Σπάνια	<1	1-3	3-5	Καθημερινά
Αλκοολούχα ποτά (ml/ημέρα, 100 ml=12g αιθανόλης)	<300	300	400	500	600	>700 η 0

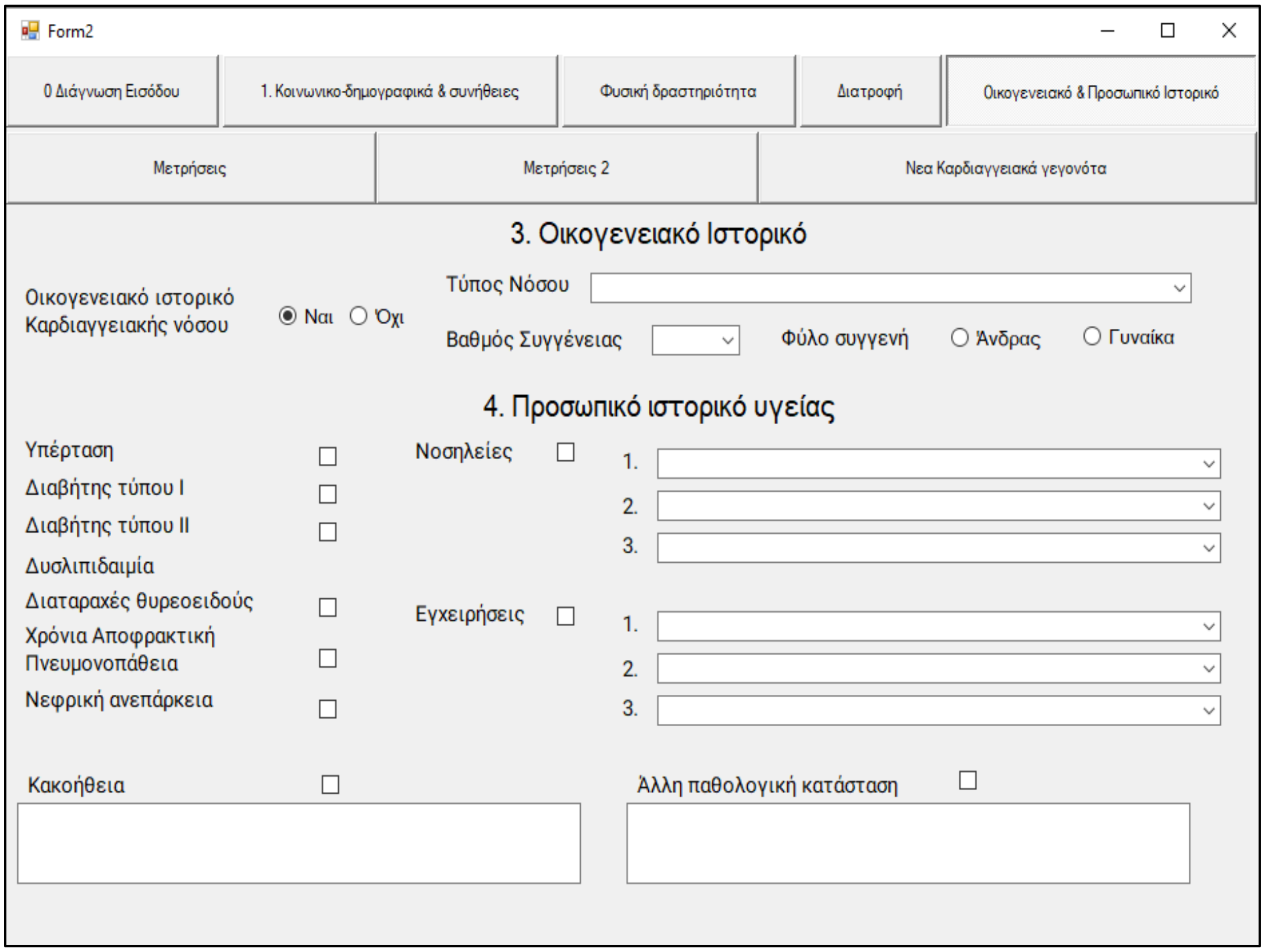
Form2
0 Διάγνωση Εισόδου
1. Κοινωνικο-δημογραφικά & συνήθειες
Φυσική δραστηριότητα
Διατροφή
Οικογενειακό & Προσωπικό Ιστορικό
Μετρήσεις
Μετρήσεις 2
Νεα Καρδιαγγειακά γεγονότα
3. Οικογενειακό Ιστορικό
Οικογενειακό ιστορικό Καρδιαγγειακής νόσου
Ναι
Όχι
Τύπος Νόσου
Βαθμός Συγγένειας
Φύλο συγγενή
Άνδρας
Γυναίκα
4. Προσωπικό ιστορικό υγείας
Υπέρταση
Διαβήτης τύπου I
Διαβήτης τύπου II
Δυσλιπιδαιμία
Διαταραχές θυρεοειδούς
Χρόνια Αποφρακτική Πνευμονοπάθεια
Νεφρική ανεπάρκεια
Νοσηλείες
1.
2.
3.
Εγχειρήσεις
1.
2.
3.
Κακοήθεια
Άλλη παθολογική κατάσταση

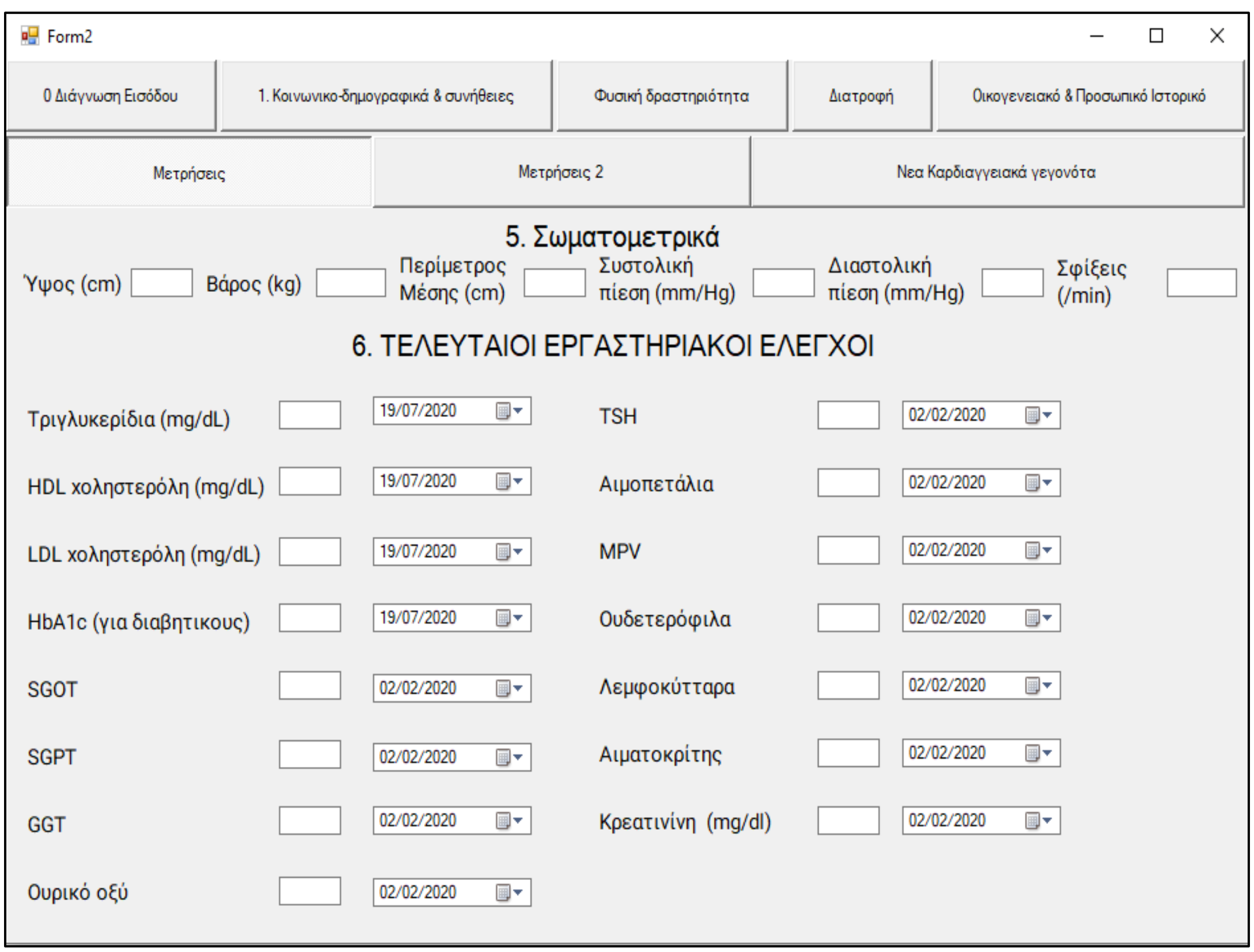
Form2
0 Διάγνωση Εισόδου
1. Κοινωνικο-δημογραφικά & συνήθειες
Φυσική δραστηριότητα
Διατροφή
Οικογενειακό & Προσωπικό Ιστορικό
Μετρήσεις
Μετρήσεις 2
Νεα Καρδιαγγειακά γεγονότα
5. Σωματομετρικά
Ύψος (cm)
Βάρος (kg)
Περίμετρος Μέσης (cm)
Συστολική πίεση (mm/Hg)
Διαστολική πίεση (mm/Hg)
Σφίξεις (/min)
6. ΤΕΛΕΥΤΑΙΟΙ ΕΡΓΑΣΤΗΡΙΑΚΟΙ ΕΛΕΓΧΟΙ
Τριγλυκερίδια (mg/dL) 19/07/2020
HDL χοληστερόλη (mg/dL) 19/07/2020
LDL χοληστερόλη (mg/dL) 19/07/2020
HbA1c (για διαβητικους) 19/07/2020
SGOT 02/02/2020
SGPT 02/02/2020
GGT 02/02/2020
Ουρικό οξύ 02/02/2020
TSH 02/02/2020
Αιμοπετάλια 02/02/2020
MPV 02/02/2020
Ουδετερόφιλα 02/02/2020
Λεμφοκύτταρα 02/02/2020
Αιματοκρίτης 02/02/2020
Κρεατινίνη (mg/dl) 02/02/2020

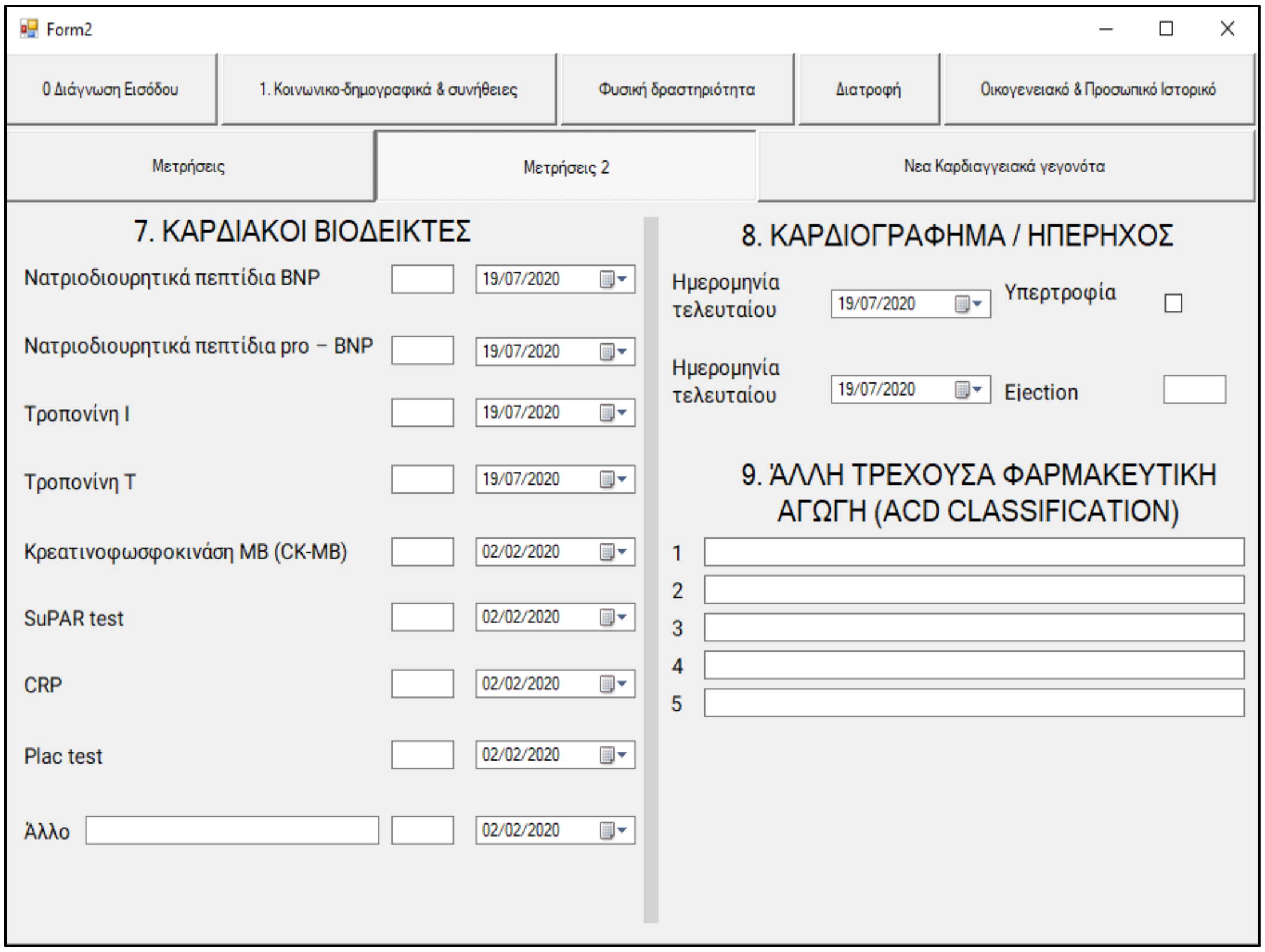
Form2
0 Διάγνωση Εισόδου
1. Κοινωνικο-δημογραφικά & συνήθειες
Φυσική δραστηριότητα
Διατροφή
Οικογενειακό & Προσωπικό Ιστορικό
Μετρήσεις
Μετρήσεις 2
Νεα Καρδιαγγειακά γεγονότα
7. ΚΑΡΔΙΑΚΟΙ ΒΙΟΔΕΙΚΤΕΣ
Νατριοδιουρητικά πεπτίδια BNP 19/07/2020
Νατριοδιουρητικά πεπτίδια pro – BNP 19/07/2020
Τροπονίνη I 19/07/2020
Τροπονίνη T 19/07/2020
Κρεατινοφωσφοκινάση MB (CK-MB) 02/02/2020
SuPAR test 02/02/2020
CRP 02/02/2020
Plac test 02/02/2020
Άλλο 02/02/2020
8. ΚΑΡΔΙΟΓΡΑΦΗΜΑ / ΗΠΕΡΗΧΟΣ
Ημερομηνία τελευταίου 19/07/2020
Υπερτροφία
Ημερομηνία τελευταίου 19/07/2020
Ejection
9. ΆΛΛΗ ΤΡΕΧΟΥΣΑ ΦΑΡΜΑΚΕΥΤΙΚΗ ΑΓΩΓΗ (ACD CLASSIFICATION)
1
2
3
4
5

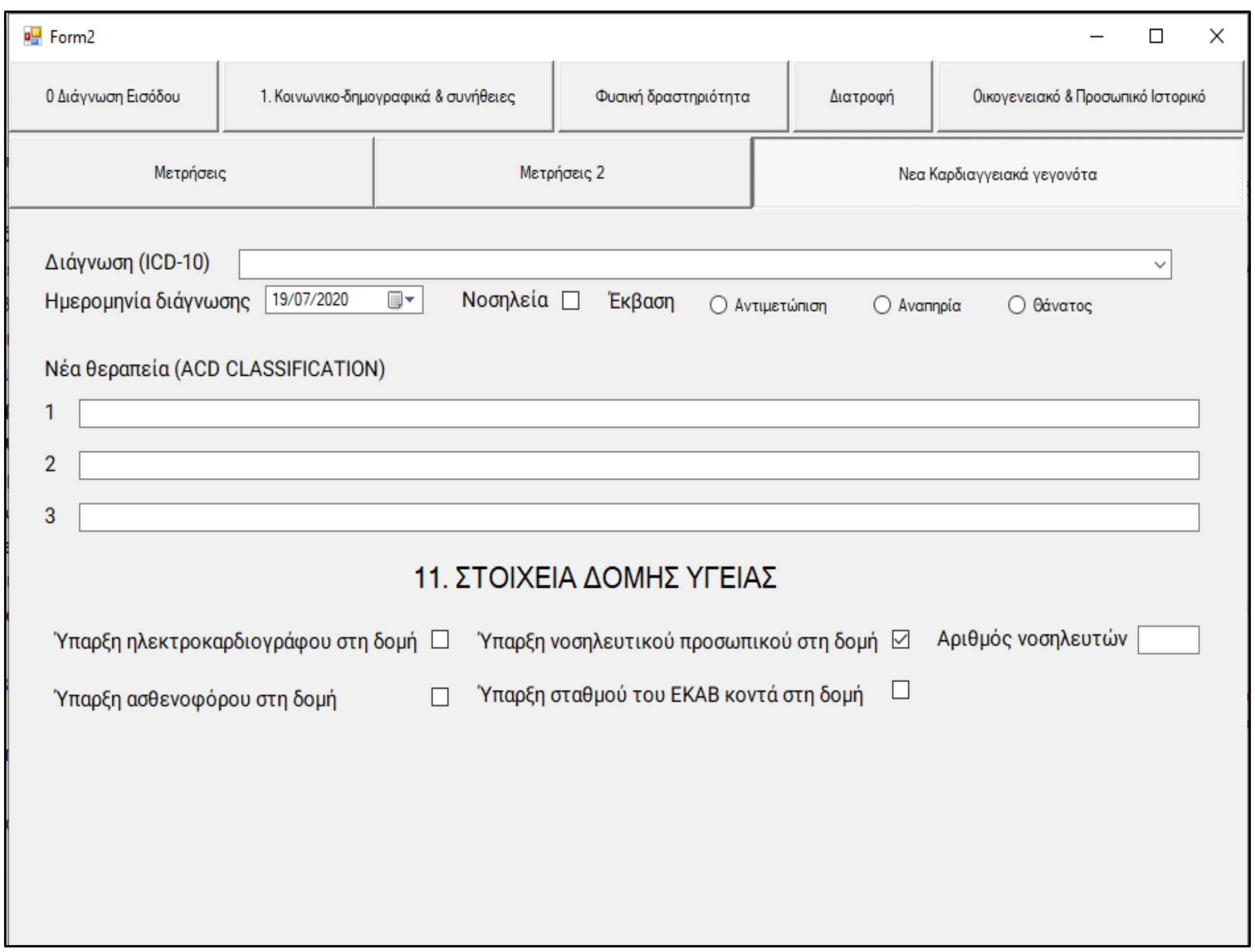
Form2
0 Διάγνωση Εισόδου
1. Κοινωνικο-δημογραφικά & συνήθειες
Φυσική δραστηριότητα
Διατροφή
Οικογενειακό & Προσωπικό Ιστορικό
Μετρήσεις
Μετρήσεις 2
Νεα Καρδιαγγειακά γεγονότα
Διάγνωση (ICD-10)
Ημερομηνία διάγνωσης 19/07/2020
Νοσηλεία
Έκβαση
Αντιμετώπιση
Αναπηρία
Θάνατος
Νέα θεραπεία (ACD CLASSIFICATION)
1
2
3
11. ΣΤΟΙΧΕΙΑ ΔΟΜΗΣ ΥΓΕΙΑΣ
Ύπαρξη ηλεκτροκαρδιογράφου στη δομή
Ύπαρξη νοσηλευτικού προσωπικού στη δομή
Αριθμός νοσηλευτών
Ύπαρξη ασθενοφόρου στη δομή
Ύπαρξη σταθμού του ΕΚΑΒ κοντά στη δομή
